# Analysis of Risk Determinants of Neonatal Mortality in the Last Decade: A Systematic Literature Review (2013–2023)

**DOI:** 10.3390/pediatric16030059

**Published:** 2024-08-12

**Authors:** Msatfa Nabila, Aziz Baidani, Yassmine Mourajid, Milouda Chebabe, Hilali Abderraouf

**Affiliations:** 1 Laboratory of Health Sciences and Technologies, Higher Institute of Health Sciences, Hassan First University of Settat, BP 555, Settat 26000, Morocco; y.mourajid@uhp.ac.ma (Y.M.); milouda.chebabe@uhp.ac.ma (M.C.); abderraouf.hilali@uhp.ac.ma (H.A.); 2 Laboratory of Agrifood and Health, Faculty of Sciences and Techniques, Hassan First University of Settat, BP 577, Settat 26000, Morocco; aziz.baidani@uhp.ac.ma

**Keywords:** neonatal mortality, risk factors, newborn, socioeconomic factors, clinical factors, healthcare access and quality, COVID-19 impact, systematic review

## Abstract

This study aimed to analyze the determinants of neonatal mortality over the last decade (2013–2023), identifying key factors that have influenced neonatal outcomes both before and during the COVID-19 pandemic. Utilizing a systematic literature review approach guided by the PRISMA method, this study evaluates 91 papers collected from indexed databases such as Scopus, PubMed, and Web of Science. The review encompasses studies conducted globally, offering insights into the evolution of neonatal mortality research and the impact of the COVID-19 crisis on neonatal health. The analysis revealed a complex array of risk determinants, categorized into socioeconomic factors, clinical factors, and healthcare access and quality. Notable factors include rural versus urban healthcare disparities, prenatal and postnatal care quality, and the influence of healthcare infrastructure on neonatal outcomes. This study highlights the shifting focus of neonatal mortality research in response to global health challenges, including the pandemic. The findings underscore the need for multidisciplinary approaches to address neonatal mortality, emphasizing the importance of enhancing healthcare systems, improving maternal education, and ensuring equitable access to quality care. Future research should explore the long-term effects of the COVID-19 pandemic on neonatal health and investigate the efficacy of interventions in diverse healthcare settings.

## 1. Introduction

Infant, neonatal, and maternal mortality rates are considered among the benchmarks commonly used to assess the health status of the population of a given society. These rates are considered as significant health indicators of a country’s level of socioeconomic development and quality of life. In any program to improve public health, reducing the neonatal mortality rate has always been a major focus and remains a major concern of any government. For this reason, it is considered an important goal of the Sustainable Development Goals (SDGs).

Global efforts to reduce these mortality rates have been led by the World Health Organization (WHO) and the United Nations. These initiatives have significantly reduced childbirth-related mortality rates worldwide. Nevertheless, the desirable reductions projected by the Millennium Development Goals have not been achieved.

While maternal mortality has decreased by 45% compared with the Millennium Development Goals reference rate [1], neonatal deaths still represent a very high rate, with 44% of infant mortality [2]. In other words, 2.8 million newborns die each year. These deaths occur in the first month after birth. It should be noted that most of these deaths occur in developing countries [2].

Several initiatives that are part of national or international programs have demonstrated their effectiveness in reducing neonatal mortality, but they remain below the expectations of civil society. The complexity of the subject, the multiplicity of causes, and the diversity of socioeconomic environments hinder the progress toward a significant reduction in these deaths and generally lead to very disparate results between countries.

Research across various global contexts has identified several key determinants of neonatal mortality. Factors such as birth order and interval, maternal age, residence area, the newborn’s gender, perceived neonate size, delivery method, seasonal birth timing, and maternal education play critical roles. Studies like those by Ezeh et al. (2014) [3] and Mekonnen et al. (2013) [4] have demonstrated that higher mortality risks are associated with younger mothers, rural locations, male newborns, and shorter intervals between births. Preventative measures, including maternal vaccinations like Tetanus Toxoid Injections, and enhanced maternal education are shown to significantly reduce neonatal mortality rates.

Furthermore, macroeconomic factors and healthcare accessibility have been highlighted as pivotal in influencing neonatal outcomes. Murad et al. (2023) [5] showed that in Bangladesh, improved healthcare expenditure and skilled birth attendance contributed to lowering mortality rates, suggesting that similar policies could benefit other developing countries. In the context of LMICs, Hazel et al. (2023) [6] and Hambisa et al. (2023) [7] found that small or preterm infants are at a higher mortality risk, while interventions such as Kangaroo Mother Care can offer a substantial reduction in preterm neonatal deaths. These findings underscore the importance of multifaceted strategies to address the complex determinants of neonatal mortality.

Nevertheless, in the post-COVID-19 context, it has become crucial to delve into the causes of infant mortality in order to devise updated strategies that address the issue effectively. An examination of the existing research in this domain is a prerequisite for such an endeavor. The culmination of this research will be synthesized into a comprehensive review article, with a specific focus on scrutinizing the factors identified as determinants of neonatal mortality. This analysis will lay the groundwork for developing interventions tailored to the current global health landscape.

This study seeks to chronicle the progression of identified factors leading to neonatal mortality and to suggest potential interventions that could significantly mitigate it. Additionally, this research will pinpoint existing gaps in knowledge to steer future inquiries toward areas most in need of exploration and understanding.

To achieve this study’s primary goals, a meticulous systematic literature review was conducted, analyzing 91 scientific articles published in peer-reviewed journals over the past decade, from 2013 to 2023. This review utilized the PRISMA (Preferred Reporting Items for Systematic Reviews and Meta-Analyses) method to identify and select data, ensuring a comprehensive and structured approach [8]. The search encompassed multiple internationally recognized indexed databases, such as Scopus, Web of Science, PubMed, Springer, Science Direct, and JSTOR, to aggregate a wide array of research findings [9]. The use of the PRISMA method underpins this research, ensuring a systematic and reproducible approach for data extraction and analysis, thereby enhancing the review’s credibility [10,11].

The structure of this paper is as follows: the “Materials and Methods” Section details the research approach, data selection, and analysis methods. The “Results” Section provides a bibliometric analysis and in-depth findings related to neonatal mortality research. In the “Discussion” Section, the risk factors of neonatal mortality are presented. Finally, the “Conclusion” Section encapsulates this study’s findings, highlighting its theoretical and practical implications while also indicating directions for future research.

## 2. Materials and Methods

### 2.1. Methodological Approach

In the “Materials and Methods” Section, we describe our use of a systematic literature review (SLR) as a methodological approach. SLRs are noted for their rigorous structure, which aids in synthesizing research comprehensively and with minimal bias, making them particularly beneficial in fast-evolving fields such as medicine and nursing [12,13]. We employed the PRISMA method for its robust framework, which helps ensure transparent reporting through a systematic process and a checklist to guide the review. This approach is instrumental in identifying research gaps and laying the groundwork for future studies, while also recognizing the need for critical assessment of SLRs to avoid over-reliance on their findings [14].

**Figure 1:**
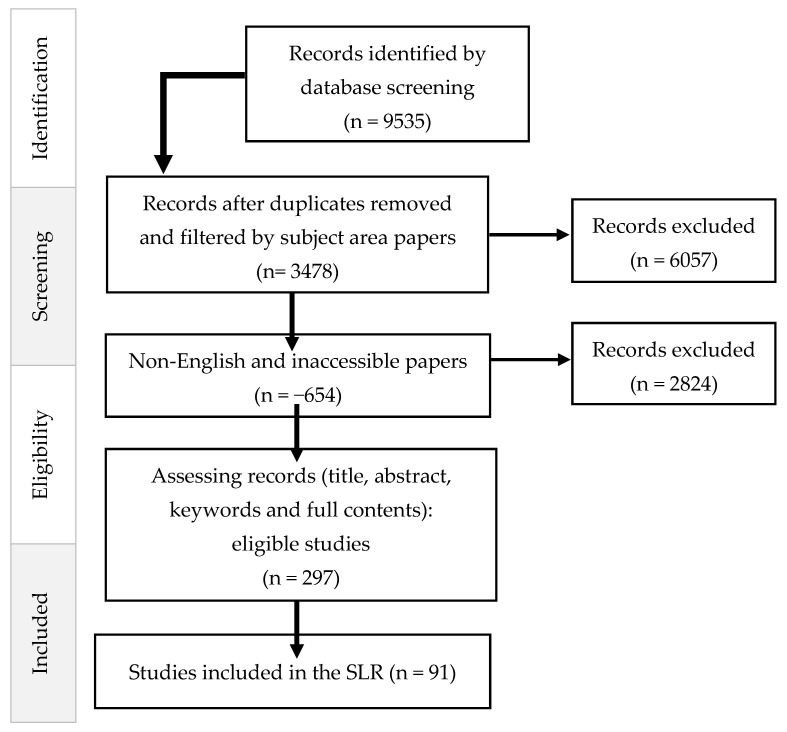
PRISMA research protocol.

Our study methodology is based on the PRISMA (Preferred Reporting Items for Systematic Reviews and Meta-Analyses) guidelines, as depicted in Figure 1. This approach was specifically chosen because of its effectiveness in exploring the determinants of neonatal mortality risk during the last decade (2013–2023), especially within the post-COVID-19 context. Our research focused on English-language articles from leading databases, such as Scopus, Web of Science (WoS), PubMed, Springer, Science Direct, and JSTOR, to ensure broad scientific acceptance and relevance. The inclusion criteria ensured that all articles were indexed in Scopus and WoS, adding to the reliability of our data sources. The PRISMA framework supports a thorough and impartial review of the literature, directing a systematic process of gathering and evaluating relevant studies. Figure 1 clearly outlines our review strategy, from the initial identification of sources through to the final selection of studies, facilitating an investigation of neonatal mortality risk factors.

### 2.2. Data Selection

The data selection process, as part of the PRISMA research protocol depicted in Figure 1, began with a database search that identified 9535 records. From these, duplicates were removed, and the records were filtered by subject area, resulting in 3478 papers. Papers that were not in English or were inaccessible were then excluded, totaling 654, leaving 2824 records to be assessed further. This assessment, which involved reviewing titles, abstracts, keywords, and full content, narrowed the pool to 297 eligible studies. Ultimately, 91 studies met the criteria and were included in the systematic literature review (SLR). The flowchart in Figure 1 visually summarizes the step-by-step process of the data selection, from the initial identification of records to the final selection of studies for inclusion in this review. The database selection step aims to find the most appropriate and high-quality research works related to our research. We tried to obtain a complete and exhaustive database and develop a solid foundation for our systematic literature review. Our research was carried out in English, and the articles cited in our work appear in the most recognized databases: Springer, Science Direct, Scopus, Web of Science, Google Scholar, PubMed, and JSTOR.

Our starting point was the relevance of the articles. Firstly, we examined the titles, abstracts, and keywords; at this level, the articles that appeared to be not relevant to this study were eliminated. Then, duplicate articles were removed to avoid double counting an article in our analysis. The remaining articles were selected based on their importance and rigor to ensure the inclusion of the most interesting work. Each selected article was thoroughly read and then carefully analyzed. This was accomplished using the following inclusion and exclusion criteria (See Table 1).

Table 1 outlines the inclusion and exclusion criteria, along with the keywords used in the data selection process for the systematic literature review. The inclusion criteria specified the type of documents considered including the following: literature review articles, empirical research articles, meta-analyses, systematic literature reviews, and conference articles. These documents were required to focus on this study’s objectives, addressing risk factors of neonatal mortality, and to be published in English. The exclusion criteria dismissed any theses and dissertations, as well as any articles that did not broadly focus on neonatal mortality. The keywords used to locate relevant articles included “infant mortality”, “neonatal mortality”, “neonatal mortality and risk factors”, “health services”, “prematurity”, “pregnancy”, and “delivery”. These terms were searched within the article title, abstract, and keywords to ensure relevance to the research scope. The table also lists the databases that were scanned for potential articles, which are Scopus, Web of Science (WoS), PubMed, Springer, Science Direct, and JSTOR. These databases were chosen for their extensive coverage of peer-reviewed scholarly literature and their recognition in the academic community.

### 2.3. Data Analysis and Synthesis

**Figure 2:**
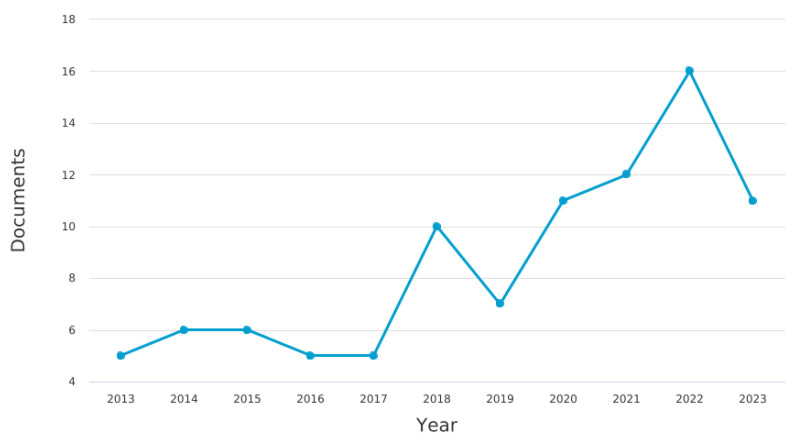
Documents by year.

**Figure 3:**
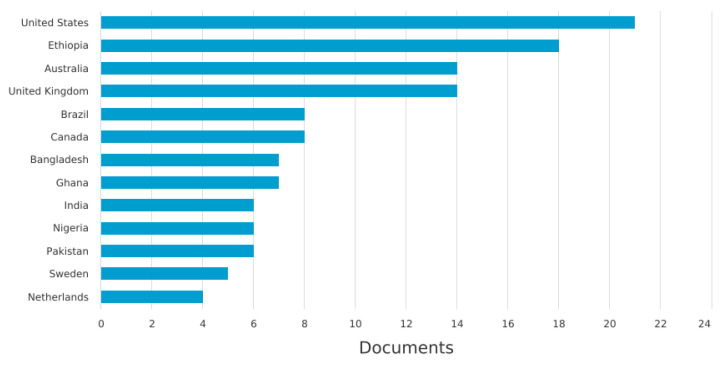
Documents by country.

**Figure 4:**
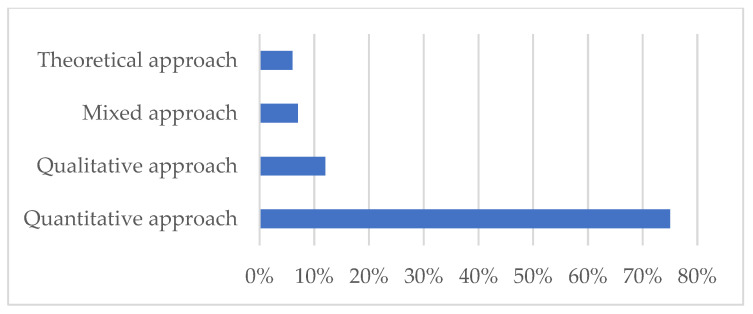
Methodology of the papers.

In the data analysis and synthesis phase of our study, we initially undertook a bibliometric analysis to explore the distribution and characteristics of the literature on neonatal mortality. This analysis included examining the number of documents by year (Figure 2), which helps understand the evolution of research interest over time, and assessing the global contribution to the literature by country (Figure 3), which highlights geographical research disparities. Additionally, we looked into the methodologies used across the papers (Figure 4) to obtain a sense of the research designs and approaches that are prevalent in the field. We also categorized the documents by type (Table 2) and by subject area (Table 3) to further dissect the landscape of neonatal mortality research, offering insights into the variety of perspectives and disciplines involved in studying this critical issue.

**Figure 5:**
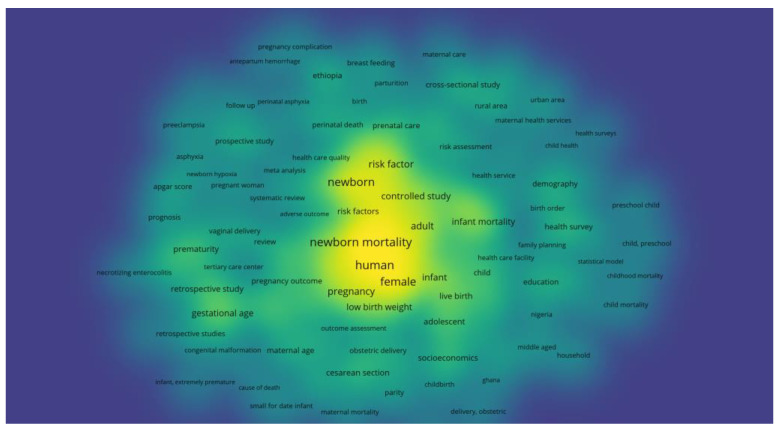
Density visualization of the main risk factors of neonatal mortality.

**Figure 6:**
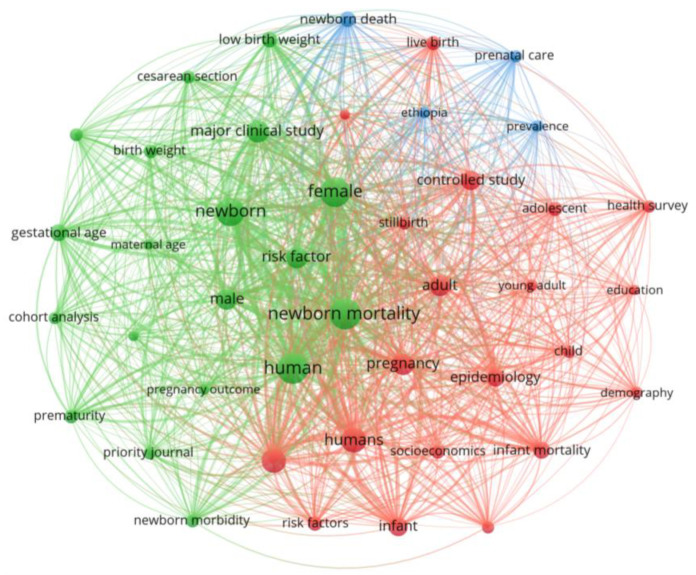
Thematic network analysis of the main risk factors of neonatal mortality.

To pinpoint the primary factors influencing neonatal mortality during the period from 2013 to 2023, we conducted a more detailed analysis of the data extracted from the 91 selected articles. Using VOSviewer 1.6.20, a software tool for constructing and visualizing bibliometric networks [15], we analyzed occurrences and keywords within the literature (Figure 5 and Figure 6). This step allowed us to map out the most prominent terms and concepts related to neonatal mortality, providing a visual representation of the research field’s thematic structure and dynamics.

Furthermore, we scrutinized the main findings of 20 papers that are directly related to our subject, as outlined in Table 4. This in-depth examination was critical for identifying the core themes and results that emerged from the most relevant studies. Finally, we synthesized the primary risk factors of neonatal mortality identified in our review and presented them in Table 5. This synthesis integrates the various determinants and insights, offering a consolidated overview of the factors that have been associated with neonatal mortality risk within the last decade, and setting the stage for potential interventions and future research directions.

## 3. Results

Moving into the Results Section, we begin by presenting the findings from our bibliometric and descriptive analysis.

### 3.1. Bibliometric Analysis

#### 3.1.1. Year of Publication

In conducting a bibliometric analysis of the literature from the past decade (2013–2023), we identified 91 articles that met our selection criteria on the subject of neonatal mortality. Our review of the annual publication trends, as depicted in Figure 2, reveals that the number of studies has fluctuated over the years, with a marked increase in research interest and publication frequency in recent years.

The year 2022 witnessed the highest volume of published articles, totaling 17, indicating a significant peak in scholarly activity. This is closely followed by the years 2021 and 2023, each with 12 publications, and 2020 with 13 articles, demonstrating sustained research focus on neonatal mortality. Earlier years like 2018 and 2019 also contributed a considerable number of articles, with 10 and 8, respectively. Conversely, the years 2013, 2016, 2015, and 2017 saw fewer publications, ranging from two to four articles, which suggests a more modest level of research interest during those years. Nonetheless, the overall trajectory indicates a pronounced increase in the number of publications, especially in the last three years, underscoring the growing academic engagement with the issue of neonatal mortality (see Figure 2). This ascending trend in the number of publications over the last decade highlights the escalating academic and clinical attention toward improving neonatal outcomes and understanding the risk determinants of neonatal mortality.

#### 3.1.2. Documents by Country

Figure 3 reveals the distribution of neonatal mortality research output by country, showing a disparity that may reflect each nation’s research capacity, economic status, and the urgency of neonatal mortality within their context.

The United States leads with the highest number of published articles (n = 21). Despite a relatively low neonatal mortality rate, the United States saw an increase from 3.49 to 3.58 deaths per 1000 live births from 2021 to 2022. Following the U.S., Ethiopia has made a significant contribution to the literature. With an infant mortality rate of 29.524 per 1000 live births in 2023, Ethiopia (n = 18) has shown a declining trend, yet the rate remains high. Other African nations like Ghana (n = 7) and Nigeria (n = 6) are also notable for their contributions despite high neonatal mortality rates, which are not specified in the provided data but are known to be among the highest globally. Bangladesh, another country with a significant neonatal mortality rate of 31 per 1000 live births, is represented in the research output. Australia (n = 14), with a low and declining infant mortality rate of 2.676 per 1000 live births in 2024, shows a strong research interest in neonatal mortality. The United Kingdom follows (n = 14), also demonstrating a commitment to research with an infant mortality rate of 3.251 per 1000 live births in 2024. Brazil’s research output is considerable (n = 8). The country’s infant mortality rate was 11.039 deaths per 1000 live births in 2023, indicating a decline from the previous year, yet highlighting a continued need for research in this area.

The data portrayed in Figure 3, alongside these infant mortality rates, underscores the complex interplay between a country’s research focus and the neonatal health challenges it faces. Countries with higher infant mortality rates contribute important research, but the volume may not always correspond to the magnitude of their public health issues. Conversely, nations with lower mortality rates, such as the United States, Australia, and the U.K., lead in research output, potentially reflecting greater resources and capacity for neonatal mortality research.

#### 3.1.3. Methodologies Used and Research Fields

Figure 4 displays the methodologies utilized in the 91 neonatal mortality papers, represented by a bar graph that categorizes the approaches into theoretical, mixed, qualitative, and quantitative. The graph reveals that the majority of the research, 75%, is based on quantitative methods, indicating a strong preference for statistical and numerical analysis in this field. Qualitative methods account for 12% of the research, highlighting a smaller yet significant portion of studies that focus on observational and interpretive data. Mixed methods, which combine both quantitative and qualitative approaches, are used in 7% of the papers, demonstrating an integrated approach in a minority of studies. Lastly, theoretical approaches, which likely involve hypothesis and concept development without empirical testing, are the least common at 6%, pointing to a lesser emphasis on purely conceptual research within the reviewed literature.

Research into neonatal mortality causes typically takes place in institutional settings such as hospitals and clinics, or through public health services at the municipal, city, district, or regional levels. The selected scientific articles predominantly focus on hospital-based studies, often employing case-study methodologies. Investigations at the regional or municipal levels are also common, with a few studies being conducted in cities to examine the broader factors affecting neonatal mortality within specific demographic contexts. This allows local managers to assess the overall situation and make informed decisions for their jurisdictions.

Notably, fewer studies are concentrated on hospital support centers and private clinics, which are often associated with higher-quality health services and do not commonly represent low-income socioeconomic groups that are at risk for neonatal mortality. Research in these settings might not provide a comprehensive view of the risk factors, potentially leading to skewed results and diminishing the relevance of other variables that influence neonatal mortality. To address these gaps and provide a comprehensive review of the knowledge surrounding neonatal mortality, our study analyzes 91 articles related to neonatal mortality risk factors. The authors of these articles hail from various nationalities, ensuring a global perspective on the issue is represented in our research.

#### 3.1.4. Documents by Type

The Table 2 provides a breakdown of the types of documents included in the systematic literature review along with their frequency and percentage representation among the 91 documents analyzed (Table 2). The majority of the documents are articles, comprising 78 of the total and accounting for 86% of the dataset. Reviews are the second most common document type, with eight instances making up 9% of the total. Book chapters and conference papers are less frequent, each representing 2% and 1% of the documents, respectively, with two book chapters and one conference paper included. Short surveys and letters are the least common, each with a single occurrence, contributing 1% to the dataset. This categorization reflects a strong preference for full articles in the research on neonatal mortality, with other document types being less prevalent in the literature.

#### 3.1.5. Documents by Subject Area

Table 3 categorizes the documents included in the systematic literature review by their respective fields, showing both the absolute number of documents and their relative frequencies as percentages of the 91 documents analyzed.

Medicine is the most represented subject area with 59 documents, comprising 65% of the total, indicating a predominant focus on medical aspects of neonatal mortality. The second most common category is Multidisciplinary, with seven documents making up 8% of the total, reflecting studies that encompass multiple fields of research. Biochemistry, Genetics, and Molecular Biology are represented by four documents, accounting for 4% of the total, while Social Sciences have three documents, making up 3%. Subject areas like Immunology and Microbiology, Environmental Science, Agricultural and Biological Sciences, Nursing, and Pharmacology, Toxicology, and Pharmaceutics each contribute one document, equating to 1% of the total for each field. These figures collectively amount to 100%, demonstrating the interdisciplinary nature of neonatal mortality research with a strong emphasis on medical research.

The bibliometric analysis of neonatal mortality research provides a detailed snapshot of the field’s evolution and current state. Publications have increased over the past decade, reflecting growing scholarly attention. Research contributions span across the globe, with the highest output from the United States and notable studies from various other countries, such as Ethiopia, Australia, the U.K., and Brazil, highlighting the worldwide concern for neonatal mortality. Quantitative methods dominate the research approaches, although qualitative, mixed, and theoretical frameworks are also present, suggesting a methodological diversity in the studies conducted. Articles constitute the majority of research outputs, followed by reviews and a smaller proportion of book chapters, conference papers, and other documents. Medicine emerges as the primary subject area, with significant contributions from multidisciplinary fields and specialties such as biochemistry, genetics, and social sciences. This diverse range of methodologies and disciplines underscores the complexity of neonatal mortality as a research topic and the interdisciplinary efforts required to address it.

### 3.2. Detailed Results

Delving into the detailed results, we go beyond mere bibliometric data to thoroughly examine the content and conclusions of the selected papers. This deeper phase of analysis leverages the insights gained from keywords and occurrences within the 91 chosen articles, utilizing VOSviewer to visualize and interpret the research landscape (see Figure 5 and Figure 6). Additionally, we conducted an in-depth analysis of the principal findings from 20 articles that are closely related to our central inquiry into the determinants of neonatal mortality across various global contexts (Table 4). This allowed us to extract and synthesize the core themes and determinants identified in the literature, providing a nuanced understanding of the factors influencing neonatal mortality rates.

The synthesis of the main findings from 20 concentrated papers on neonatal mortality reveals various determinants and interventions across diverse contexts. Factors such as preterm birth complications, neonatal infections, asphyxia, low birth weight, and socioeconomic conditions are commonly identified contributors to neonatal death. In Ghana, [16] highlighted the effectiveness of home visits for essential newborn care in reducing neonatal mortality rates. The authors of [17], in their study spanning 67 countries, pointed out that delayed breastfeeding initiation and poor delivery conditions are significant risk factors. In the United Kingdom, [18] found a strong association between low Apgar scores and higher mortality due to anoxia or infection, especially in preterm infants. Promising interventions include Kangaroo Mother Care, as emphasized by Hambisa et al. (2023) [7] in Ethiopia, and the improvement of healthcare systems’ quality and accessibility. In Kenya, [21] underscored the importance of prenatal care education, qualified birth attendants, and adequate emergency services during delivery. In Brazil, [22] stressed the need for socioeconomic improvements, timely access to health services, and high-quality prenatal care.

Studies from South Asia and Sub-Saharan Africa by [23] recommend higher parental education, better economic status, and improved neonatal care. In Taiwan, [30] suggested that giving birth in tertiary facilities and avoiding labor transfers could significantly reduce neonatal mortality. Lastly, in Bangladesh, [31] highlighted the critical role of biological, demographic, and socioeconomic factors, along with an efficient healthcare system. Overall, the body of research underscores the complex, multifactorial nature of neonatal mortality, suggesting that a combination of healthcare interventions, socioeconomic enhancements, and improved maternal and neonatal care practices is essential for effectively reducing mortality rates.

As we progress in our analysis, we employ VOSviewer to conduct a keyword examination of the 91 selected articles, aiming to identify and visualize the main risk factors associated with neonatal mortality. This tool allows us to map the most prominent terms used in the research, providing a density visualization that helps us understand the most discussed topics and how frequently they are mentioned across the studies.

In Figure 5, the density visualization, or “heat map”, showcases the concentration of keywords within the literature. Larger and more centrally located terms such as “newborn”, “risk factor”, “low birth weight”, and “neonatal mortality” are indicative of the primary concerns and focal points within the field. The prominence of these terms suggests that they are common threads in neonatal mortality research, with a high occurrence across various studies. Other notable keywords include “pregnancy”, “human”, “female”, “infant”, and “gestational age”, which are also significant but perhaps slightly less central than the most dominant terms. This visualization also highlights related areas of interest, such as “breastfeeding”, “maternal care”, “pregnancy complications”, and “preterm birth”, which are crucial factors in the context of neonatal health.

The density of the keywords across the visualization indicates the relative importance and interconnectivity of these terms within the research landscape. Terms that are closer together are likely to be related or appear together in the literature, suggesting areas of research that may share common methodologies or findings. This visualization serves as a strategic tool to identify the most studied risk factors and to guide future research toward less explored but potentially critical areas within the neonatal mortality domain.

Figure 6 offers a thematic network analysis of the main risk factors associated with neonatal mortality, which was created using VOSviewer. This network visualization maps the relationships between the keywords from the 91 selected articles, illustrating how various terms and themes are interconnected within the research.

In the network, each node represents a keyword, and its size reflects the frequency of the term’s occurrence in the literature, with larger nodes indicating more common terms. The lines, or edges, connecting the nodes demonstrate the strength of the association between terms, with thicker lines showing stronger relationships.

Central to the network are terms like “newborn”, “risk factor”, “human”, and “infant mortality”, which form the core of the research focus. Surrounding these are other important nodes like “low birth weight”, “gestational age”, “pregnancy”, and “prematurity”, which are also significant factors in the studies but may have varying degrees of direct connection to the central terms.

This thematic network also reveals clusters of terms, with different colors representing different themes or categories of interconnected concepts. For example, we can observe a cluster around “socioeconomics” and “infant mortality”, indicating a body of research that examines the impact of socioeconomic status on neonatal outcomes. Other clusters might focus on clinical factors such as “cesarean section” and “birth weight”, or demographic factors like “female”, “male”, and “maternal age”.

Overall, Figure 6 provides a visual representation of the complex interplay between different factors influencing neonatal mortality. By identifying these connections, researchers and policymakers can better understand the multifaceted nature of neonatal mortality and the areas where interventions might be most effective.

## 4. Discussion

In the discussion of this synthesis, emphasis has been placed on certain factors due to the availability of reliable data and their alignment with established models and hypotheses in the field. The case studies analyzed provide a snapshot of the varied scenarios affecting neonatal mortality, although they do not encompass every possible situation. Nevertheless, they offer insights into the predominant factors influencing infant death rates, even though the list of variables identified is not exhaustive.

The interactions among these variables are complex and often interrelated, though exploring these relationships is beyond the scope of this analysis. For clarity, we categorize our analysis into three main categories as follows: factors pertaining to the newborn, the mother, and the healthcare system.

The table synthesized from the VOSviewer analysis (Table 5) neatly organizes the main risk factors of neonatal mortality into determinant categories. This categorization underscores the complexity of the risk factors, which span from socioeconomic conditions to clinical and healthcare service-related variables. The multifaceted nature of these determinants reflects the breadth of factors that must be considered when addressing neonatal mortality.

Table 5, which synthesizes the main risk factors of neonatal mortality, presents a comprehensive view of the various determinants that have been identified in the literature as influencing neonatal outcomes. These factors are categorized into socioeconomic factors, clinical factors, and healthcare access and quality.

Socioeconomic factors, as noted by authors such as [31,33], include the environment where the mother and child reside, such as rural or urban areas, the family’s social and economic status, education level, household conditions, family planning practices, parity, and overall maternal welfare. These factors contribute to disparities in neonatal mortality rates, with studies showing higher mortality in poorer and less educated households.

Clinical factors encompass a range of medical and biological conditions that can affect a newborn’s chances of survival. Some research has discussed how antepartum hemorrhage, asphyxia, variations in birth weight, gestational age, complications arising from prematurity, cesarean section delivery, maternal age, and the need for neonatal intensive care can all impact neonatal mortality [16,32]. The presence of congenital malformations also plays a significant role.

Healthcare access and quality are crucial determinants of neonatal mortality. Studies by [20,21] emphasize that hospital admission, the quality-of-care facilities, healthcare services provided, maternal care, maternal health services, and the availability of tertiary care centers are all pivotal to reducing neonatal mortality. The quality of the health system directly affects neonatal outcomes, with better-resourced facilities leading to lower rates of neonatal death.

Regarding factors related to the newborn, as elucidated by [18,28], the Apgar score is a critical assessment tool for determining the immediate health of the newborn, with lower scores associated with higher risks of neonatal and infant death. Asphyxia is a leading cause, with studies indicating that effective oxygen therapy and immediate neonatal care can significantly improve outcomes.

For health system-related factors, the research points to the vital role of healthcare quality in neonatal survival. As highlighted by authors like [22,29], access to skilled healthcare, professional birth attendants, and immediate postnatal care is essential. Improved infrastructure, adequate staffing, and investment in healthcare organizations are recommended to enhance neonatal care and reduce mortality rates.

Lastly, factors related to the mother’s general situation, as discussed by [26,30], involve socioeconomic conditions that affect perinatal and neonatal mortality. These include the mother’s age at delivery, pregnancy care, educational level, and socioeconomic status. Studies indicate that improvements in these areas can lead to better neonatal outcomes. Neonatal mortality is influenced by a complex interplay of socioeconomic, clinical, and healthcare system factors. Addressing these determinants through targeted interventions and systemic improvements is key to reducing neonatal mortality and improving outcomes for newborns globally.

For newborn-related factors, clinical assessments like the Apgar score are crucial for determining a neonate’s initial health, particularly their circulatory, respiratory, and neurological states. Perinatal asphyxia is a major cause of neonatal death, with other causes including pulmonary immaturity and maternal–fetal infection. Authors like [18,28] emphasize the importance of immediate care, including oxygen therapy and neonatal resuscitation, to mitigate these risks.

Healthcare system factors play a significant role in neonatal outcomes. High-quality healthcare services are essential for reducing mortality, as illustrated by studies from [20,21], showing that professional care during delivery in healthcare facilities correlates with lower mortality rates. Inadequate care, particularly in low-resource settings as noted in Sub-Saharan Africa by [35], contributes significantly to neonatal deaths. Geographic location and access to care are also crucial, as highlighted by research in Myanmar and Cambodia cited by [31]. Maternal factors such as socioeconomic status, education level, and living conditions have been demonstrated to impact neonatal mortality. A study carried out in France has shown that mortality rates vary according to socio-professional category, suggesting that higher socioeconomic backgrounds facilitate better prenatal care and, consequently, better neonatal outcomes [36,37]. The interactivity of these factors, as discussed by [38,39], highlights the need for a holistic approach to addressing neonatal mortality, considering gender, birth weight, gestational age, and maternal age, among other variables.

The introduction of the Sustainable Development Goals (SDGs) focuses on improving newborn health, aiming to reduce infant mortality rates significantly. Addressing disparities in care quality and ensuring all population segments receive adequate care are essential steps towards achieving these goals. Authors like [39] advocate for improved prenatal services to decrease potential life loss, underlining the importance of continuous system improvement and quality care provision to combat neonatal mortality effectively.

## 5. Conclusions

The bibliometric analysis of the neonatal mortality literature over the last decade reveals a field that is not only expanding but also diversifying, with contributions coming from all over the globe. The United States leads in research output, but significant insights also come from Ethiopia, Australia, the United Kingdom, and Brazil, among others, reflecting a universal dedication to understanding and solving the challenges of neonatal mortality.

Quantitative research methods are prevalent, yet there is a valuable presence of qualitative, mixed-method, and theoretical studies, indicating an array of approaches to tackle neonatal mortality issues. Peer-reviewed articles are the predominant form of communication in the field, supplemented by systematic reviews, book chapters, and conference papers. Medicine stands as the primary subject area, yet the importance of interdisciplinary studies is evident with notable inputs from biochemistry, genetics, social sciences, and more. When synthesizing the detailed results from keyword analyses and thematic assessments, several determinant categories emerge, including socioeconomic factors, clinical factors, and healthcare access and quality. These findings highlight the multifaceted nature of neonatal mortality and underscore the necessity of addressing a wide range of determinants to improve neonatal outcomes.

In conclusion, the comprehensive analysis of neonatal mortality research paints a picture of a dynamic and intricate field. While advancements have been made, the persistence of neonatal mortality underscores the need for continued research and intervention. Effective strategies must account for clinical, socioeconomic, and healthcare system factors and must be sensitive to the diverse contexts in which neonatal mortality occurs. Future efforts should focus on leveraging the strengths of multidisciplinary approaches and international collaboration to forge solutions that are as complex and nuanced as the problem itself. Addressing neonatal mortality requires a concerted global effort, one that combines the knowledge gained from extensive research with actionable policies and practices to safeguard the lives of the youngest and most vulnerable members of society.

### 5.1. Theoretical and Practical Implications

The theoretical and practical implications of the research on neonatal mortality are substantial, influencing both academic understanding and real-world health outcomes. The body of research on neonatal mortality contributes significantly to theoretical implications within the field. The complexity of the risk factors identified highlights the inadequacy of simplistic models and promotes a more comprehensive, multidimensional understanding of neonatal mortality, recognizing it as a multifaceted issue with a web of interrelated determinants. The diversity in methodologies—spanning quantitative, qualitative, and mixed methods—calls for theoretical frameworks that can accommodate and synthesize different types of data and findings. Additionally, the global nature of the research, with studies conducted in a variety of cultural, economic, and health system contexts, underscores the necessity for theoretical constructs that are adaptable to diverse regional specifics, allowing for nuanced analyses and applications that are both globally informed and locally relevant.

On the practical side, the implications are equally profound and actionable. Research emphasizing the critical role of prenatal and postnatal care points to the need for targeted interventions that can substantially lower neonatal mortality rates, as demonstrated in the work of [16,21]. The correlation between healthcare access, service quality, and neonatal outcomes suggests that enhancing healthcare infrastructure, especially in under-resourced areas, can lead to significant improvements in neonatal survival, as observed by [24,29]. These findings inform policy and program development, advocating for comprehensive approaches that address healthcare directly while also considering broader socioeconomic factors. Additionally, the research advocates for the expansion of education and training for healthcare providers and parents, recognizing the importance of knowledge and preparedness in improving neonatal health outcomes. Lastly, the call for stronger, more integrated healthcare systems reflects a consensus on the need for a cohesive approach to obstetric and neonatal care to effectively reduce mortality rates, a sentiment echoed by [37] and other researchers.

### 5.2. Research Limitations and Futures Directions

The research presented has certain limitations that should be acknowledged. Focusing exclusively on the last decade may inadvertently overlook significant research conducted before 2013. This temporal boundary may omit crucial developments and insights from earlier studies, which could enhance the understanding of trends and patterns in neonatal mortality. Nevertheless, the research spanning the last decade offers a unique advantage, as it encompasses studies conducted both before and after the onset of the COVID-19 crisis (2013–2019 and 2019–2023, respectively). This allows for a comparison of neonatal mortality trends and factors in pre-pandemic and pandemic/post-pandemic contexts. The ability to analyze changes and challenges brought on by COVID-19 provides a valuable dimension to the research, offering insights into how such global health crises can influence neonatal mortality rates and the effectiveness of health systems in responding to them.

Looking ahead, there is a clear need for further empirical research to delve deeper into less explored areas. A qualitative empirical study examining the role of healthcare services and quality management within hospitals could shed light on their impact on neonatal mortality risk, particularly in under-researched contexts. Such a study would offer in-depth insights into hospital operations, leadership, staff competencies, and institutional policies that could influence neonatal outcomes. Additionally, a quantitative empirical study using a questionnaire to measure the impact of all identified factors on neonatal mortality risk could provide a broader understanding of the relative importance and interplay of these factors. By combining both qualitative and quantitative approaches in future research, a more nuanced understanding of the determinants of neonatal mortality could be achieved, informing both policy and practice to improve newborn health outcomes globally.

## Figures and Tables

**Table 1:** Inclusion and exclusion criteria and keywords used in data selection.

Inclusion criteria	Document type	Literature review article, empirical research article, meta-analysis, systematic literature review, and conference article.
	Objectives	All studies that address risk factors of neonatal mortality.
	Language	Articles published in English on the topics of neonatal mortality.
Exclusion criteria	Type of document	Thesis and dissertations.
	Objectives	Any article that does not focus on neonatal mortality in the broad sense.
Keywords	“Infant mortality”, “neonatal mortality”, “neonatal mortality and risk factors”, “health services”, “prematurity”, “pregnancy”, “delivery”	
Scanned items	Article title, abstract, keywords.	
Database	Scopus, Web of Science (WoS), PubMed, Springer, Science Direct, and JSTOR,	

**Table 2:** Documents by type.

Documents	Frequency	%
Article	78	86%
Review	8	9%
Book chapter	2	2%
Conference paper	1	1%
Short Survey	1	1%
Letter	1	1%
	91	100%

**Table 3:** Documents by subject area.

Subject Area	Documents	Frequency (%)
Medicine	59	65%
Multidisciplinary	7	8%
Biochemistry, Genetics, and Molecular Biology	4	4%
Social Sciences	3	3%
Immunology and Microbiology	2	2%
Environmental Science	1	1%
Agricultural and Biological Sciences	1	1%
Nursing	1	1%
Pharmacology, Toxicology, and Pharmaceutics	1	1%
	91	100%

**Table 4:** Key insights from 20 concentrated studies on neonatal mortality risk factors.

Author	Country	Method Study Population	Sample	Results: Validated Factors	Perspectives: Variables Reducing Neonatal Mortality
Kirkwood et al. [16]	Ghana, the Brong Ahafo region	Cluster randomized controlled trial/pregnancies that ended in live birth or stillbirth between November 2008 and December 2009 from 98 new hint areas.	15,200 live births	Home visits to perform necessary care can reduce the risk of neonatal mortality.	Home visits for promoting essential newborn care practices and treating or referral of sick babies can reduce neonatal mortality rates.
Boccolini et al. [17]	67 countries in all continents	Ecological panel study on 67/ live births from 67 countries. This study used secondary data from 67 countries obtained from Demographic and Health Surveys.	N = 1000	- Delayed breastfeeding initiation. - Poor delivery and birth care conditions. - Complications of prematurity. - Birth asphyxia.	Helping mothers to initiate breastfeeding in this sensitive period, with mother and newborn on the alert.
Iliodromiti et al. [18]	Royaume-Uni (Ecosse)	Population-based cohort study/ singleton live births in women with a gestational age at delivery between 22 and 44 weeks.	N = 2307 cases	A low Apgar score at 5 min was strongly associated with the risk of neonatal and infant death due to anoxia or infection, with an additional association with hyaline membrane disease in preterm infants.	The authors did not suggest any recommendations.
Grünebaum et al. [19]	United States	Retrospective study/infant births and deaths linked to the Centers for Disease Control and Prevention in the United States from 2006 to 2009.	4 million births in the United States each year	- Home deliveries by a midwife were associated with a considerably greater risk of total infant death than hospital midwife births. - The rate of total and early neonatal mortality for home births remained higher, as did the risks for women of 41 weeks or more and women with a first birth. - Deliveries outside of hospitals greatly raised the chance of infant mortality.	Physicians and other health care providers have a professional responsibility to understand, identify, and address the underlying cause of patients’ desire to give birth outside of the hospital by providing evidence-based compassionate hospital care, improving the hospital setting, treating obstetrical interventions, and providing excellent hospital care.
Fernandes et al. [20]	Mozambique	Longitudinal subnational study ((Demographic and Health Survey (2003 and 2011) and Multiple Indicator Cluster Survey (2008)) data /children under 5 and newborns.	26,464 children in 2000, 20,936 children in 2005, and 10,697 children in 2010	-Facility-based birth assistance. -The overall density of the public sector health workforce and the density of maternal and child health nurses and coverage of facility-based birth assistance were strongly associated with reduced neonatal mortality.	Improving public sector health human resources and increasing coverage of institutional births, as well as increasing public spending on health.
Yego et al. [21]	Kenya	Retrospective case-control study/fetal deaths and live births.	Cases were early fetal and neonatal deaths (n = 200) and controls were newborns alive immediately before and after the cases (n = 400)	- Birth attendant qualifications; gestational age; and number of prenatal visits. - Maternal complications at birth (hemorrhage and dystocia). - Congenital malformations and low five-minute Apgar scores.	- Education of mothers on prenatal consultation, screening, monitoring, and management of maternal conditions during the prenatal period should be strengthened. - The presence of a physician at every delivery and emergency admission is critical to ensuring early infant survival and avoiding potential risk factors for mortality. - The development of protocols for the management of newborns and regular audits based on criteria to prevent early neonatal mortality.
Batista et al. [22]	Brazil	Case control study/ population of live births in 2008.	1772 controls for 412 cases	- Socioeconomic level. - Mother with a history of fetal loss. - Time to find health services before delivery. - Absence of a health care professional at the time of delivery. - Congenital malformations. - Prematurity. - Regional and intra-national inequality.	- Adequate and high-quality prenatal care, guaranteed access to a maternity hospital before birth, timely care for the parturient woman, and competent hospital care for the mother and baby. - Investment in health-care organization, such as expanding the number of beds in critical care units and neonatal intensive care units.
Ahmed et al. [23]	South Asia/Sub-Saharan Africa	Multi-country prospective cohort study from 2012 to 2016/women of reproductive age (15–49 years) in Sub-Saharan Africa and South Asia.	278,186 pregnancies and 263,563 births	- Perinatal asphyxia. - Severe neonatal infections. - Complications of prematurity.	-Programs in sub-Saharan Africa and South Asia should intensify efforts to reduce mortality rates. -Access to high-quality, standardized, population-based prospective studies to quantify the burden, causes, and timing of deaths, including neonatal deaths. Training and advocacy workshops.
Garcia et al. [24]	Brazil	Cohort study: The study was based on a historical cohort of live births, developed as part of the epidemiological surveillance activities of the Municipal Health Secretariat in 2016 in Brazil/live births.	15,879 live births	-Risk factors related to behavior and health service use (number of prenatal visits, type of health service, and type of delivery). -Apgar score and biological risk factors (sex, identified malformation, birth weight, weeks of pregnancy, identified malformation, type of pregnancy, maternal age).	The authors did not suggest any recommendations.
de Souza et al. [25]	Brazil	Case-control study/ neonatal deaths in Foz do Iguassu (Brazil) from 2012 to 2016.	25,563 births	-Factors associated with neonatal death were congenital fetal anomalies; low birth weight, first minute Apgar score less than 7, and prematurity.	The implications for practice that can be drawn from the study include the following: - Prenatal surveillance should be increased, and hospital treatment for pregnant women and newborns should be prioritized. - Future studies on the quality of prenatal, delivery, and early postpartum care, preterm risk factors, the influence of cross-border patients on birthing indicators, and the epidemiology of birth anomalies on the international border are needed.
Ferraz et al. [26]	Portugal	Retrospective case-control study/medical records of all vaginal deliveries between January 2012 and December 2016.	Two control groups including 1802 spontaneous deliveries and 909 forceps-assisted deliveries	-The risk of soft tissue trauma, cephalohematoma, jaundice, intensive phototherapy, and transitory brachial plexus injury.	The authors did not suggest any recommendations.
Barquiel et al. [27]	Spain	Retrospective observational study/newborns of women with gestational diabetes.	Neonates born to 3413 women with gestational diabetes	-Factors associated with gestational diabetes mellitus (severity of hyperglycemia at diagnosis, glycemic control, and gestational weight gain). -Weight of the newborn. -Gestational weight gain of the mother was a factor for neonates born small for gestational age (SGA).	-The prevalence of SGA should be added to the rate of LGSs and cesarean sections in future studies of DG-related complications. - Diabetes screening methods for mothers whose fetuses are growing slowly should be developed. - Once GDM is identified, dietary changes and obstetric care should be followed to lower the risk of SGA complications.
Grünebaum et al. [28]	United States	Retrospective study/ infant births and deaths for 2010 to 2017.	CDC10-related infant birth and death records for 2010 to 2017	-The location of the birth. -The nature of the birth attendant.	-Neonatal mortality can be significantly optimized by planning hospital births.
Guinsburg et al. [29]	Brazil	Retrospective study/ population of live births and deaths from 0 to 27 days in 2004–2013 in the region of Sao Paulo.	All children born in the state of São Paulo to mothers residing in the state in 2004–2013 were included	- Respiratory disorders. - Congenital malformations. - Infections. - Perinatal asphyxia. - Birth year, mother education level, marital status, maternal age, and multiple pregnancies. - Prenatal/delivery care. - Delay in obtaining appropriate care.	- Access to skilled health care should be prioritized to minimize newborn mortality in São Paulo.
Chang et al. [30]	Taiwan	Retrospective cohort study/ all live-born infants of a single birth, with a birth weight between 500 and 1499 g and a gestational age of 22 completed weeks, born between 2011 and 2014.	4560 very low birth weight infants (VLBW)	Gender, low birth weight, gestational age of the infant, and maternal age. Non-medical assistance and hospitalization of an early birth (of gestational age infants). Birth in a non-tertiary hospital and postnatal transfer. Intrapartum transfer of pregnant women. Lack of coordination between obstetric and neonatal services.	-Tertiary facility birth may reduce neonatal mortality rates. -A policy of delivering low body weight infants in medical centers could potentially further reduce neonatal and infant mortality. Avoiding the transfer of women during the intrapartum period. - Avoiding transfer of infants at gestational age. - Provide neonatal intensive care units in rural areas.
Islam and Biswas [31]	Bangladesh	Mixed study (cross-sectional and meta-analysis)/	Sample of 21 developing countries including Bangladesh	-Micro: biological/demographic factors, socioeconomic status, health care system, cultural practices, and technologies were key determinants of neonatal death and the interactions among its variables. -Macro: the socioeconomic condition of a country, the effectiveness. and efficiency of health care systems.	-Higher education for parents. -Improving the economic status of parents. -Superior birth rank. -Delivery at an age above 19 years. -Care during the neonatal period can all reduce neonatal mortality.
Eyeberu et al. [32]	Ethiopia	A cross-sectional study/ newborns.	834 neonates	-Antepartum hemorrhage, pregnancy-induced hypertension, and type of pregnancy. - Low birth weight, perinatal asphyxia, and early onset neonatal sepsis.	- Improve care for all newborns, with a specific emphasis on high-risk neonates, and focus on factors that affect neonatal survival to minimize neonatal death.
Hambisa et al. (2023) [7]	Ethiopia	Systematic review and meta-analysis of studies delineating preterm mortality and associated factors.	17 studies	Preterm mortality associated with neonatal sepsis, respiratory distress syndrome, and perinatal asphyxia. Kangaroo Mother Care significantly reduced mortality.	Kangaroo Mother Care; improving preterm neonatal healthcare system; and community-based healthcare strategies.
Alabi et al. [33]	Sub-Saharan Africa	Literature review of the impact of COVID-19 on maternal and child health.	Not specified	The COVID-19 pandemic increased maternal and child mortality, highlighting the importance of antenatal care and preparedness for health crises.	Prioritizing women’s antenatal care and developing policies for health crises and emerging diseases.
Sampurna et al. [34]	Indonesia	National survey data analysis of 10,838 live-born infants from singleton pregnancies in 2017.	10,838 live-born infants	Lack of postnatal care and delivery complications (other than prolonged labor) associated with increased neonatal death; low birth weight infants at higher risk.	Improving quality and services of public hospitals and equitable distribution of healthcare services.

**Table 5:** Synthesis of the main risk factors of neonatal mortality.

Categories of Factros	Factors
Socioeconomic factors	Rural area and urban area. Social status. Socioeconomics. Education. Household. Family planning. Parity. Maternal welfare.
Clinical factors	Antepartum hemorrhage. Asphyxia. Birth weight. Gestational age. Low birth weight. Prematurity. Cesarean section. Maternal age. Neonatal intensive care. Premature birth. Prenatal care. Vaginal delivery. Congenital malformations.
Healthcare access and quality	Hospital admission. Health care facility. Health care quality. Health service. Maternal care. Maternal health services. Tertiary care center.

## Data Availability

No new data were created or analyzed in this study. Data sharing is not applicable to this article.
